# Faster permutation inference in brain imaging

**DOI:** 10.1016/j.neuroimage.2016.05.068

**Published:** 2016-11-01

**Authors:** Anderson M. Winkler, Gerard R. Ridgway, Gwenaëlle Douaud, Thomas E. Nichols, Stephen M. Smith

**Affiliations:** a Oxford Centre for Functional MRI of the Brain, University of Oxford, Oxford, UK; b Department of Statistics & Warwick Manufacturing Group, University of Warwick, Coventry, UK; c Wellcome Trust Centre for Neuroimaging, UCL Institute of Neurology, London, UK

**Keywords:** Permutation tests, Negative binomial distribution, Tail approximation, Gamma distribution, Generalised Pareto distribution, Low rank matrix completion, Pearson type III distribution

## Abstract

Permutation tests are increasingly being used as a reliable method for inference in neuroimaging analysis. However, they are computationally intensive. For small, non-imaging datasets, recomputing a model thousands of times is seldom a problem, but for large, complex models this can be prohibitively slow, even with the availability of inexpensive computing power. Here we exploit properties of statistics used with the general linear model (GLM) and their distributions to obtain accelerations irrespective of generic software or hardware improvements. We compare the following approaches: (i) performing a small number of permutations; (ii) estimating the *p*-value as a parameter of a negative binomial distribution; (iii) fitting a generalised Pareto distribution to the tail of the permutation distribution; (iv) computing *p*-values based on the expected moments of the permutation distribution, approximated from a gamma distribution; (v) direct fitting of a gamma distribution to the empirical permutation distribution; and (vi) permuting a reduced number of voxels, with completion of the remainder using low rank matrix theory. Using synthetic data we assessed the different methods in terms of their error rates, power, agreement with a reference result, and the risk of taking a different decision regarding the rejection of the null hypotheses (known as the resampling risk). We also conducted a re-analysis of a voxel-based morphometry study as a real-data example. All methods yielded exact error rates. Likewise, power was similar across methods. Resampling risk was higher for methods (i), (iii) and (v). For comparable resampling risks, the method in which no permutations are done (iv) was the absolute fastest. All methods produced visually similar maps for the real data, with stronger effects being detected in the family-wise error rate corrected maps by (iii) and (v), and generally similar to the results seen in the reference set. Overall, for uncorrected *p*-values, method (iv) was found the best as long as symmetric errors can be assumed. In all other settings, including for familywise error corrected *p*-values, we recommend the tail approximation (iii). The methods considered are freely available in the tool PALM — Permutation Analysis of Linear Models.

## Highlights

• Permutation methods can be accelerated through additional statistical approaches.
• Six approaches are described and assessed.
• Methods can be 100 times faster than in the non-accelerated case.
• Recommendations are provided for various common scenarios.

## Introduction

Permutation tests allow exact control of error rates, with minimal assumptions. However, permutation tests are computationally intensive. For small, non-imaging datasets, recomputing a model thousands of times is seldom a problem, but for imaging applications, that involve testing at thousands of spatial points (voxels, vertices, faces, edges), large models that involve many subjects, multiple measurements, pointwise (voxelwise) regressors, spatial statistics, as well as other sources of complexity, even with the availability of inexpensive computing power, the same procedure can be prohibitively slow. Strategies to accelerate the process include the use of efficient or optimised code, the use of parallel, multi-threaded, or distributed computing, and the use of graphics processing units (GPUs) (for example applications of the latter, see Eklund et al., 2012, Eklund et al., 2013, Hernández et al., 2013). While these methods are attractive for increases in speed, none reduce the amount of tasks that effectively need to be executed, and the improvements in speed happen through more efficient use of resources available, or through the introduction of yet more resources. At a time in which Moore's law (Moore, 1965) approaches physical limits (Waldrop, 2016), alternative methods to expedite computation are expected to gain prominence.

Here we exploit properties of the statistics themselves and their distributions, which could be used to accelerate the evaluation of the test in order to accept or reject the null hypothesis in a fraction of the time that otherwise would be needed with a large number of permutations. The main tenet of these approaches is to obtain a reduction of the number of actual computations that need to be performed, such that acceleration can be obtained in addition to, or irrespective of, generic improvements of software or hardware. In particular, we discuss the following approaches: (i) performing a small number of shufflings (with no other change from the usual case of permutation tests); (ii) estimation of the *p*-value as a parameter of a negative binomial distribution; (iii) fitting of a generalised Pareto distribution to the tail of the empirical permutation distribution; (iv) computing the *p*-values based on the expected moments of the empirical distribution, approximated from a gamma distribution; (v) direct fitting of a gamma distribution to the empirical distribution; and (vi) shuffling of a reduced number of points (e.g., voxels), with completion of the remainder using low rank matrix theory. Details of each are provided in the Theory section.

Very few of such acceleration strategies have been investigated or used in brain imaging. The tail approximation was considered by Ge et al. (2012) for an imaging genetics application in which, due to the sheer volume of data, conventional permutation tests were not considered feasible. A variant of many possible algorithms for low rank matrix completion was proposed by Hinrichs et al. (2013). The fitting of a gamma distribution without the need for permutations was proposed recently for a range of statistics by Minas and Montana (2014). For inference for support-vector machine problems, Gaonkar and Davatzikos (2012) suggested an analytical approximation to the permutation distribution of each component of the hyperplane that separates classes. Here we aim to study, evaluate, and in some cases propose, solutions that can accelerate permutation tests for the general linear model (GLM), considering aspects that are specially relevant to imaging, such as the multiplicity of tests and the use of spatial statistics. In particular, we make the following main contributions: (I) show how a connection between Pillai's trace and the popular univariate *t* statistic allows the direct computation of the *p*-values from the permutation distribution, even without performing actual permutations, (II) use the moments of the empirical permutation distribution for the fit of a gamma distribution, and (III) propose a novel low rank matrix completion algorithm, writing the test statistic as the product of two matrices that can be sampled sparsely, and allowing exact recovery of what otherwise would be an approximation.

### Overview of the paper

**Fig. 1:**
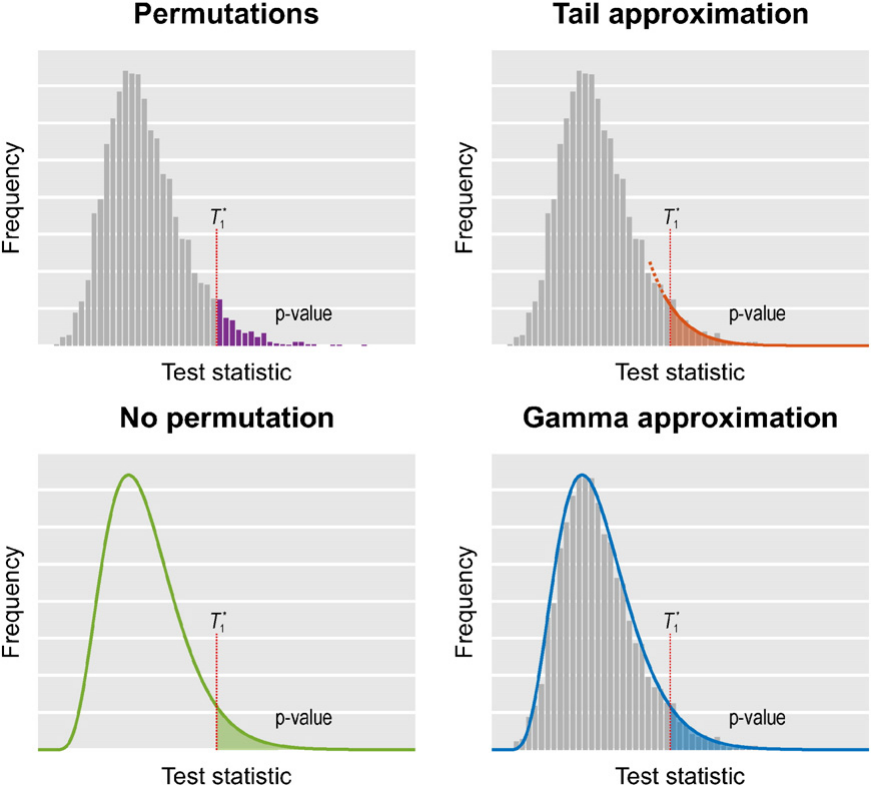
With permutations (i.e., any number of rearrangements, the use of the negative binomial distribution, or the low rank matrix completion), the *p*-value is the fraction of the test statistics obtained after permuting that are higher than in the unpermuted *T* ≡ *T*_1_^⁎^. In the tail approximation, the tail of the permutation distribution is subjected to the fit of a generalised Pareto distribution (GPD), from which the *p*-values are computed. In the method in which no permutations are performed, the first three moments of the permutation distribution are computed from data and model, and these moments are used to fit a gamma distribution (Pearson type III) from which the *p*-values are computed. In the gamma approximation, the moments of the empirical permutation distribution are used for the fit of the gamma distribution. The fig. is merely illustrative: the actual fit uses the cumulative distribution function, such that histograms are not constructed in practice, hence the fit does not depend on binning.

In the Theory section we begin by briefly reviewing the uni- and multivariate GLM, their assessment using permutation tests, and introduce the notation used throughout the paper. The six different acceleration strategies are then presented in sequence, followed by certain aspects related to spatial statistics and multiple testing correction in the context of these methods. In the Evaluation and Results sections we assess the performance of these different methods on both synthetic and real data. In the Discussion we provide recommendations for general circumstances. A summary of the acceleration strategies is provided in Table 1. Fig. 1 illustrates four of them.

## Theory

### Notation and general aspects

At each spatial point of an image representation of the brain, consider a general linear model (GLM) (Scheffé, 1959) expressed as:

(1) Y=Mψ+ε

where **Y** is the *N* × *K* matrix of observed data, with *N* observations of *K* distinct (possibly non-independent) variables, **M** is the full-rank *N* × *R* design matrix of explanatory variables (i.e., effects of interest and possibly nuisance effects), ***ψ*** is the *R* × *K* matrix of regression coefficients, and ***ε*** is the *N* × *K* matrix of random errors. Estimates for the regression coefficients can be computed as $\hat{\psi }=M^{+}Y$, where the superscript (^+^) denotes a generalised inverse. One is generally interested in testing the null hypothesis that a contrast of regression coefficients is equal to zero, i.e., $H_{0}:C'\psi D=0$, where **C** is an *R* × *S* full-rank matrix of *S* contrasts of coefficients on the regressors encoded in **M**, 1 ⩽ *S* ⩽ *R* and **D** is a *K* × *Q* full-rank matrix of *Q* contrasts of coefficients on the dependent, response variables in **Y**, 1 ⩽ *Q* ⩽ *K*; if *K* = 1 or *Q* = 1, the model is univariate. Once the hypothesis has been established, **Y** can be equivalently redefined as **YD**, such that the contrast **D** can be omitted for simplicity, and the null hypothesis stated as $H_{0}:C'\psi =0$. Another useful simplification is to consider a transformation of the model into a partitioned one:

(2) Y=Xβ+Zγ+ε

where **X** is the matrix with regressors of interest, **Z** is the matrix with nuisance regressors, and ***β*** and ***γ*** are respectively the vectors of regression coefficients. Even though such partitioning is not unique, it can be defined in terms of the contrast **C** such that the columns of **X** and **Z** are orthogonal to each other, and inference on ***β*** is equivalent to inference on **C**'***ψ*** (Beckmann et al., 2001, Smith et al., 2007, Winkler et al., 2014). A suitable, pivotal test statistic, here generically termed *T*, is computed and its significance assessed through permutations and/or sign flippings of the data, the model, the residuals, or variants of these. We sometimes use the terms *rearrangement* or *shuffling* when the distinction between permutations or sign flippings is not pertinent. The *p*-value is computed as:

(3) $$p=\frac{1}{J}\sum_{j=1}^{J}I\left(T_{j}^{*}⩾T\right)$$

where *I*(⋅) is the indicator function, *T*_*j*_^⁎^ is the test statistic observed at the *j*-th shuffling of the data, and *J* is the number of rearrangements performed, of which the first (i.e., *j* = 1) is the unpermuted case. We denote the significance level of the test as *α*. In typical cases, *J* is much smaller than the number of unique possible rearrangements allowed by the design and data, *J*^max^. The same procedure can be used with classical multivariate tests (CMV), such as MANOVA/MANCOVA or canonical correlation analysis (CCA), as well as with Non-Parametric Combination (NPC); details for both the univariate and multivariate GLM in the context of imaging are discussed in Winkler et al. (2014, 2016).

#### Resampling risk

Two methods may have similar error rates and power, yet fail to agree on which tests should have their null hypotheses rejected or retained. The *resampling risk* is a quantity that represents the probability of taking a different decision regarding the rejection or acceptance of the null hypothesis if the procedure is repeated using the same input data, but different methods (Jöckel, 1984). Compared to confidence intervals, which can be calculated for *p*-values derived from permutations through a binomial approximation (see the Section Few permutations), the resampling risk is a more generic quantity in that it provides information on the chance of reaching a different decision regarding the null hypothesis that is computable for all the different methods, including, for instance, the one in which no permutations are used.

### Acceleration methods

Nearly all of the acceleration strategies below can be applied to univariate, uncorrected pointwise tests (“pointwise” as an umbrella term encompassing voxelwise, vertexwise, facewise, as well as nodewise and edgewise graph theoretical measurements, or any other relevant imaging test). If *Q* > 1 or *K* > 1, the model is multivariate, and CMV or NPC can be considered (Winkler et al., 2016). Some of the methods can also be used with spatial test statistics, and for inferences corrected for the familywise error rate (FWER) using the distribution of the extremum statistic (see below).

#### Few permutations

Conditional on the observed data, if all possible rearrangements are performed, a permutation test is exact in that it yields results that are not based on distributional assumptions or asymptotic approximations, but rather represent the exact probability of rejecting the null hypothesis when it is true. If fewer than all possible rearrangements are performed, the *p*-value obtained is an estimate of the true and unknown *p*-value; the test continues to be exact in that the probability of obtaining an estimate $\hat{p}$ less than or equal to the significance level *α*, is *α* itself, i.e., *P*($\hat{p}$ ⩽ *α*) = *α*, provided that the level is sensibly chosen considering the discreteness of the permutation *p*-values. Thus, a simple strategy for acceleration consists in running only a small number of permutations. As indicated above, this results in an unbiased (i.e., correct on average) estimate of the *p*-value, but with higher variance (variability around the true value) than when using a large number of permutations. Confidence intervals around $\hat{p}$ can be computed using one of the various methods for Bernoulli trials, such as those proposed by Wilson (1927), Clopper and Pearson (1934) or Agresti and Coull (1998) (for a comparative review, see Brown et al., 2001). Whichever is used, fewer permutations imply wider intervals (Table 2), such that the resampling risk can be expected to increase; in the Evaluation section we assess this risk for the case of a few permutations, as well as for the other acceleration methods.

#### Negative binomial

If the permutations are performed randomly (as opposed to in some order, such as lexicographic), after a few permutations there may already be sufficient information on whether the null should be rejected, and continuation of the process narrows the confidence interval around $\hat{p}$, although with little chance of changing a decision about the rejection of the null hypothesis if the estimated *p*-value lies far from the test level *α*. The process can therefore be interrupted after some criterion has been reached. Various such criteria have been proposed (Andrews and Buchinsky, 2000, Davidson and MacKinnon, 2000, Fay and Follmann, 2002, Fay et al., 2007, Gandy, 2009, Kim, 2010, Sandve et al., 2011, Gandy and Rubin-Delanchy, 2013, Ruxton and Neuhäuser, 2013), and of particular interest is the interruption after a predefined number *n* of exceedances *T*_*j*_^⁎^ ⩾ *T* has been found. Weaker effects will quickly be exceeded after a few random shufflings, whereas stronger effects require insistence in doing more shufflings until exceedances are found. The ensuing *p*-value is the estimated parameter of a negative binomial distribution (Haldane, 1945) as $\hat{p}=\left(n-1\right)/\left(j-1\right)$, where *j* is the permutation in which *n* was reached; this does not include the unpermuted case, and once that is considered, the permutation *p*-value becomes $\hat{p}=n/j$. This method was proposed by Besag and Clifford (1991), and compared to other approaches, it is attractive for its negligible computational overhead, and for bypassing the need that *α* or any other parameter is defined beforehand. If *n* has not been reached after a sufficiently large predefined number *J* of permutations, the process can be interrupted regardless, and the *p*-value computed as in Eq. (3).

#### Tail approximation

The limiting distribution of the maximum of a set of identically distributed random variables converges to one of three well known families of distributions, under a form given by the generalised extreme value distribution (GEV) [Gnedenko (1943); for reviews, see Leadbetter et al. (1983), Davison and Huser (2015)]. More broadly, however, the tail of the distribution of an arbitrary random variable can be approximated using a generalised Pareto distribution (GPD) (Picklands, 1975). For a threshold *u* → ∞, the limiting distribution of the quantity *y* = *T* − *u*, for *T* > *u*, is *F*(*y*) = 1 − (1 − *ξy*/*σ*)^1/*ξ*^, defined for *y* > 0 and *ξy*/*σ* < 1, with parameters *ξ* (shape) and *σ* (scale).^1^ Methods to estimate the two parameters of the GPD from the observed permutation statistics include maximum likelihood, the method of moments, or the method of probability-weighted moments; all three have similar estimation efficiency for − 1/2 < *ξ* < 1/2, as typical in real world applications (Hosking and Wallis, 1987, Knijnenburg et al., 2009). Using the method of moments, the estimators of the scale and shape parameters are $\hat{\sigma }=\overset{-}{y}\left({\overset{-}{y}}^{2}/s^{2}+1\right)/2$ and $\hat{\xi }=\left({\overset{-}{y}}^{2}/s^{2}-1\right)/2$, where $\overset{-}{y}$ and *s*^2^ are respectively the sample mean and variance of the values *y* (Hosking and Wallis, 1987). Goodness of fit can be assessed with the Anderson–Darling test (Anderson and Darling, 1952, Choulakian and Stephens, 2001, Knijnenburg et al., 2009).

The algorithm proceeds as follows: a small number of permutations is initially performed, the set of test statistics *T*_*j*_^⁎^ is recorded for each image point, and initial *p*-values computed as in Eq. (3). The voxels with *p*-values above a loose, liberal significance level (such as twice the chosen *α*) remain unchanged; the others have the tail of their permutation distribution used to estimate the GPD parameters. For these, a reasonable, initial threshold *u* is the *T*_*j*_^⁎^ that defines the upper quartile of their respective permutation distribution. This threshold is iteratively increased until a good fit of the GPD is found; if a good fit is not found when the permutation distribution has been exhausted, no approximation is made, and the initial *p*-value is not modified; otherwise, a new *p*-value is computed using the tail of the GPD fitted for that voxel. For the initial permutation distribution, the unpermuted statistic (*T*_1_^⁎^) may or may not be included in the process of tail fitting, and the impact of its inclusion depends on the number of permutations used for the initial distribution, as we show in the Results section.

#### No permutation

Pillai's trace (Pillai, 1955) is a suitable statistic that can be considered to test $H_{0}$. With the partitioned model, it can be computed as T=traceY~'HXY~Y~'Y~−1, where **H**_**X**_ = **XX**^+^, $\tilde{Y}=R_{Z}Y$, **R**_**Z**_ = **I** − **ZZ**^+^, and **I** is the *N* × *N* identity matrix. Alternatively, it can be computed as *T* = trace(**H**_**X**_**UU**'), where **U** is a *N* × *K* matrix containing the *K* left singular vectors of $\tilde{Y}$ that have non-zero singular values.^2^ Let **A** ≡ **H**_**X**_ and **W** ≡ **UU**', such that *T* = trace(**AW**). For statistics that can be written in this form, with **A** and **W** being *N* × *N* symmetric matrices with mean-centered columns, the first three moments of the permutation distribution of the *N*! possible values for *T* can be computed analytically under the assumption of symmetry of the error terms (Box and Watson, 1962, Mardia, 1971, Kazi-Aoual et al., 1995). With the moments known, a gamma distribution can be fitted, from which *p*-values can be obtained without permutations. The gamma distribution is the Type III distribution in the Pearson system (Pearson, 1895); references to the classical name often appear when the distribution is parameterised with respect to its moments, although here the current name is used to keep in pace with modern terminology.

The requirement of mean-centered columns for **A** and **W** implies that the model intercept is entirely represented in **Z**, and that all columns of **X** have zero mean. This imposes a restriction on the set of designs for which this method can be considered. Simple group comparisons and correlations between continuous variables, for instance, are easily accommodated, whereas the means of individual groups are not.

When rank(**C**) = 1 and *K* = 1 (or *Q* = 1), which is by far the most commonly encountered situation, the contrast has a direction (positive or negative), but Pillai's trace is two-tailed, which in principle would seem to diminish its usefulness, and limit the uses of the above relationship to just a few situations. This is not a problem in practice: if *T* is Pillai's trace, then $\text{sign}\left(\beta \right)\sqrt{T}$ is the partial correlation coefficient, which has a monotonic relationship with, and therefore is permutationally equivalent to, the *t* statistic. Assuming that the (unknown) distribution of *t* is symmetric around zero, a *p*-value for the directional test can be computed by halving the *p*-value obtained from the gamma fit to the distribution of *T*, then subtracting the result from unity if the sign of the regression coefficient in the partitioned model (*β*) is negative. Thus, these relationships allow *p*-values that are based on the moments of the permutation distribution for Student's *t*-tests to be obtained, without doing any actual permutation.

#### Gamma approximation

Even for statistics that cannot be written in the form *T* = trace(**AW**), the fit of a gamma distribution through moment matching has potential to yield valid, useful approximations (Solomon and Stephens, 1978, Minas and Montana, 2014). This category includes the distributions of spatial statistics, as well as the distribution of the extremum statistic, which is used to control error rates for the multiplicity of tests (both are discussed below). For such statistics, a small number of permutations is performed, the first three moments (mean, variance, and skewness) are estimated from the permutation distribution, and a gamma distribution with the corresponding moments fitted, from which the *p*-values are computed. As with the tail approximation, the unpermuted statistic (*T*_1_^⁎^) may or may not be included in the initial permutation distribution (we evaluate both ways, and return to this aspect below). The gamma distribution does not have infinite support in both directions, but some test statistics do have, and sometimes the unpermuted test statistic may fall outside the support of the fitted curve. To address this issue, depending on the direction of the skewness, the respective *p*-value is replaced by either 1 or 1/*J*, i.e., the smallest attainable if no approximation is done.

#### Low rank matrix completion

The statistics computed for each permutation can be organised in a matrix **T** of size *J* × *V*, where *J* is the number of permutations and *V* is the number of image points (voxels, vertices, etc). Assuming that **T** has a low rank, only a small, random subset of its entries needs to be sampled; the missing ones can instead be recovered approximately using results from low rank matrix completion theory (Candès and Recht, 2009, Candès and Tao, 2010), with appreciable acceleration. However, despite the fact that **T** tends to have a dominant low rank component, with many small values in the eigenspectrum, it is still of full rank for statistics that are non-linear functions of the data, which is the case for nearly all the useful ones. Ignoring the end of the spectrum causes loss of information. While the rank can be recovered through the introduction of random noise with similar moments (Hinrichs et al., 2013), there is no guarantee that it will possess the same spatial structure that would preserve the distribution of spatial statistics used in imaging. There is also no guarantee that the residual noise can be characterised by the parameters of a particular distribution, which is at odds with a usable recovery of this matrix. This is the case even considering that some of the acceleration methods discussed in this paper explicitly make this assumption in different contexts.

Here we follow a different strategy: we factorise **T** into a pair of matrices that can be assembled from linear functions of the data, thus allowing **T** to be recovered *exactly*. We begin by recalling that, using the partitioned model, when rank(**C**) = 1 and *Q* = 1, a suitable statistic is the *t* statistic, such that each element of **T** is computed as $T_{\mathrm{jv}}={\hat{\beta }}_{\mathrm{jv}}{\left(X'X\right)}^{1/2}/{\hat{\sigma }}_{\mathrm{jv}}$, where ${\hat{\beta }}_{\mathrm{jv}}$ are the estimated regression coefficients for the *j*-th permutation and *v*-th voxel, and ${\hat{\sigma }}_{\mathrm{jv}}$ is the standard deviation of the respective residuals, σ^jv2=ε^jv'ε^jv/N−rankM. Thus, **T** = *κ***B** ⊙ **Σ**^[− 1/2]^, where **B** is a *J* × *V* matrix that has entries ${\hat{\beta }}_{\mathrm{jv}}$, **Σ** is a similarly sized matrix whose entries are the sums of squares of the residuals, ςjv=ε^jv'ε^jv, *κ* = (**X** ' **X**(*N* − rank(**M**)))^1/2^ is a scalar constant, ⊙ is the Hadamard (elementwise) product, and the bracketed exponent in **Σ** indicates elementwise power. In this formulation, it is **B** and **Σ** that are subjected to sparse sampling and low rank matrix completion, instead of **T** directly; the results of completion are used to compute **T** exactly, rather than approximately, provided that certain conditions are met.

Such exact matrix recovery is not possible unless at least as many entries as the degrees of freedom of the matrix, *ν*, are observed, a quantity that depends on the size and rank of the matrix to be recovered (Candès and Tao, 2010), and that should not to be confused with the degrees of freedom associated with the GLM. For a *J* × *V* matrix, *ν* = *r*(*J* + *V*) − *r*^2^, where *r* is the matrix rank. For full rank matrices, this implies observing all their entries, and doing so would not bring any speed improvement. However, provided that the matrix to be completed has rank *r* < min (*J*, *V*), then *ν* < *J* ⋅ *V*, so that not all its entries need to be seen or sampled. Moreover, if an orthonormal basis spanning the range of the matrix is known, such as its left singular vectors, complete recovery of the missing entries on any row or column can be performed using ordinary least squares regression (Troyanskaya et al., 2001), provided that, respectively, at least *r* observations are available on each row or column. If fewer are available, approximate recovery may still be possible.

Our objective is to sample some of the entries of **B** and **Σ**, fill the missing ones, and compute **T**. Although **B** and **Σ** do not need to have a matching set of entries sampled, it is convenient to do the sampling simultaneously, as both are produced from the same regression of the GLM. The number of entries that needs to be sampled depends then on which of these two matrices has the highest rank. To determine that, note that **B** can be computed as a product of a *J* × *N* and an *N* × *V* matrix. The rows and columns of each of these are determined, respectively, by the permutation and regression strategy, as shown in Table 3. With any of these strategies, the matrix product makes it clear that the upper bound on the rank of **B** is *N*. Likewise, **Σ** depends on the permutation and regression strategy, and its rank cannot be larger than the number of possible distinct pairs of *N* observations, which imposes an upper bound on the rank of **Σ** at *N*(*N* + 1)/2.

Thus we have the conditions in which not all samples are needed, that allow exact recovery of **T**, and from which an algorithm arises naturally: (I) min(*J*, *V*) > *N*(*N* + 1)/2, (II) orthonormal bases spanning the range of **Σ** are known, and (III) for each permutation *j*, at least as many tests (e.g., voxels) as the rank of **Σ** are observed. For condition (I), the number *N* of subjects should ideally not be chosen based on speed considerations, but rather on statistical power and costs associated with data collection, and can be considered fixed for an experiment. The number *V* of points in an image is typically very large, such that this condition is trivially satisfied. The number *J* of permutations, however, can be varied, and should be chosen so as to satisfy (I). For condition (III), at least as many voxels than the rank of **Σ** are randomly sampled. For condition (II), orthonormal bases can be identified by first running a number *J*_0_ = *N*(*N* + 1)/2 of permutations using all *V* tests, and assembling initial fully sampled **B**_0_ and **Σ**_0_ matrices, which are subjected to SVD. With the two bases known, subsequent permutations *j* = {*J*_0_ + 1, … , *J*} are done using a much smaller set of voxels; the results for these are projected to the respective orthonormal bases, recovering the complete *j*-th row of **B** and **Σ** for that permutation, and hence the corresponding row of **T**. This proceeds as follows: consider the singular value decomposition **USV** ' = **B**_0_, where **U** is an *r* × *V* orthonormal basis, *r* = rank(**B**_0_), *r* < *V*. In a given permutation *j*, a (possibly random) number *v*, *r* ⩽ *v* < *V* of entries of the row ***β***_*j*_ of **B** is observed; call this 1 × *v* row ${\tilde{\beta }}_{j}$. The complete row can be recovered as $\beta_{j}={\tilde{\beta }}_{j}{\tilde{U}}^{+}U$, where $\tilde{U}$ contains the respective *v* columns of **U** that match the observed row entries. The same procedure can be applied to the rows ***ς***_*j*_ of **Σ**, using the basis derived from **Σ**_0_. **Σ** and **Σ**_0_ have only positive entries, and to minimise the effects of sign ambiguity on the recovered data (for a description of the problem, see Bro et al., 2007), the mean can be subtracted before SVD, and added back after recovery.

The full matrix **T** is never actually needed. Instead, at each permutation, its *j*-th row is computed using completion as above, and discarded after counters have been incremented (Eq. (3)). To ensure that all permutations are treated equally, the permutations *j* = {1, … , *J*_0_} can be revisited and recomputed through low rank matrix completion once the orthonormal bases for **B**_0_ and **Σ**_0_ have been obtained.

A similar strategy can be considered for cases in which rank(**C**) > 1 or *Q* > 1, for statistics other than *t*. However, to accommodate more regression coefficients for the *F*-statistic, or the various off-diagonal sums of products in the multivariate case for statistics as Wilks' *λ* or Pillai's trace, more than just two matrices would need to be sampled and filled, causing further computational costs that have potential to nullify, or even reverse, acceleration improvements. Finally, the dependence of the completion on a common design for all *V* tests does not allow for pointwise (voxelwise) regressors in the design matrix; all other acceleration methods discussed in this paper, however, allow for this possibility.

### Inference for spatial statistics

The distribution of spatial statistics, such as cluster extent (Friston et al., 1994), cluster mass (Poline et al., 1997, Bullmore et al., 1999) and threshold-free cluster enhancement (TFCE) (Smith and Nichols, 2009), can be computed using few permutations, from which *p*-values can be assessed. These can be further refined, at the tails, with a generalised Pareto distribution, or using the fit of a gamma distribution. The performance of these approaches for spatial statistics are assessed below. The negative binomial approximation cannot be used, because the permutations at each voxel are interrupted after a different number of permutations, preventing spatial statistics from being computed correctly (except for FWER, see below). Moreover, these statistics cannot be trivially written as trace(**AW**), such that the method with no permutations cannot be used either. Finally, with low rank matrix completion, while it is possible to compute these statistics after missing voxels have been filled, it is unlikely that useful improvements on speed can be obtained, as most of the time spent on spatial statistics rests on the computation of neighbourhood information. A direct, possibly non-exact, recovery of spatial statistics could be considered, though not with the proposed algorithm.

### Multiple testing correction

Controlling the FWER requires the distribution of the extremum (across tests) statistic. This means that the method in which no permutations are done cannot be used, as the extremum cannot be written as trace(**AW**). The negative binomial, as proposed, if operating individually at each test (voxel) cannot be used either: later rearrangements include fewer voxels than the initial ones, thus changing the skewness of the distribution of the extremum as the shufflings are performed. A possible workaround for the negative binomial is to interrupt the shufflings once the extremum across tests in a given permutation exceeds (a number *n* of times) the extremum in the unpermuted case; the empirical distribution of the maximum statistic obtained at this point is used for the adjustment the *p*-values. This permits also the use of spatial-statistics. A potential problem for this approach is that all voxels in an image would depend entirely on the result found for the single, most extreme test in the unpermuted case: an incidental incorrect result at that single voxel would affect the results across the whole image.

Other methods can be used directly for FWER-correction: few permutations, tail and gamma approximations, and low rank matrix completion can all be used. For the tail and gamma, the GPD and the gamma distribution are, respectively, fitted to the distribution of the extremum after a fixed, possibly small number of permutations has been performed. For the low rank matrix completion, the distribution is obtained by taking the maximum across the *V* columns of **T**, thus producing a vector of length *J* containing the extrema, from which *p*-values can be computed for all voxels in the image.

Such correction is not limited to the points within an image: under the same principles, the extremum statistic can be used to correct across multiple imaging modalities, multiple contrasts (i.e., multiple hypotheses using the same data), as well as a mixture of imaging and non-imaging data (Winkler et al., 2016), provided that the test statistic is pivotal, that is, that its asymptotic sampling distribution does not depend on unknown parameters (Winkler et al., 2014).

Controlling the false discovery rate (FDR) (Benjamini and Hochberg, 1995, Genovese et al., 2002) requires that, under the null, the distribution of the *p*-values is uniform on the interval [0 1]. This condition can be relaxed by accepting *p*-values that are valid for any significance level smaller than or equal to the proportion of false discoveries that the researcher is willing to tolerate, i.e., *α* ⩽ *q*_FDR_, which not only encompasses the original definition, but also accommodates the cases (e.g., with TFCE) in which the uniformity of the distribution of *p*-values is lost only for high *p*-values, which are typically of no interest. It should be noted, however, that from its own definition, FDR is expected to be conservative with discrete *p*-values if too few permutations are performed, which can be predicted from the original formulation, and as it has been described in the literature (Gilbert, 2005). This can be the case if some tests are found significant (the true proportion of false discoveries may be smaller than the level *q*_FDR_, due to ties), or if none is found significant (the true familywise error rate, usually weakly controlled by FDR, may be below *q*_FDR_ or even equal to zero, as the lower bound on the *p*-values, dictated by the number of permutations, may not be sufficiently small to allow any rejection).

### Algorithmic complexity

The actual time needed to perform each method depends on choices made at implementation, including programming strategies, resources offered by the programming language and the compiler, as well as the available hardware. Asymptotic bounds and memory requirements are more realistic as means to provide a fairer comparison, and a summary is shown in Table 4. Compared to an ideal method in which a very large, potentially exhaustive (*J*^max^), number of shufflings is performed, and that would have asymptotic computational complexity Θ(*NVJ*^max^), each method uses a different strategy to increase speed. Few permutations, tail and gamma approximations use small *J*. Speed is increased in the negative binomial case by means of reducing the number of shufflings based on the number *n* of exceedances needed, thus having a stochastic runtime. The no permutation case bypasses the need for permutations altogether. Compared to the others, the low rank matrix completion has lower asymptotic run time when *N* is small in relation to *V* and *J*.

As the acceleration in each of the methods is due to different mechanisms, the stage at which the increments in speed happen varies. For few permutations, as well as for tail and gamma approximations, the increases in speed happen through the use of fewer shufflings; the latter two, however, need additional time to allow the fit of a GPD or gamma distribution respectively, to the initial, permutation distribution. For FWER-corrected results, such fitting is quick, as it needs to be performed for only one distribution (of the extremum statistic); for uncorrected results, however, this process takes considerably longer, as each voxel needs its own curve fitting. The negative binomial benefits from fewer permutations, and further, benefits from a reduction in the number of tests (voxels) that need to be assessed, although there is a computational overhead due to the selection of tests that did not reach the number of exceedances and need to continue to undergo permutations. The low rank matrix completion benefits from a dramatic reduction in the number of tests that need to be done, a quantity that depends only on the number of subjects and not on the size of the images. The method in which no permutations are performed benefits from the analytical solution and, as the name suggests, the waiver of the need to permute anything.

The memory requirements also vary. For the few permutations and negative binomial, only the array of *V* elements containing the test statistic, and another of the same size for the counters to produce *p*-values are needed. For the tail and gamma approximations, the test statistics for all *J* permutations need to be stored, from which the moment matching is performed. The no permutation does not require counters. The low rank matrix completion needs two arrays of size *V* × *J*_0_ to store the values of **B**_0_ and **Σ**_0_, and two further arrays of the same size to store the orthonormal bases (at which point **B**_0_ and **Σ**_0_ are no longer needed).

## Evaluation methods

In an initial phase, we explored all methods using synthetic univariate and multivariate data and a wide variety of parameters. We assessed their performance in terms of agreement of the *p*-values with those obtained from a reference set constructed from a relatively large number of permutations, which provide information on error rates and power. In a second phase, using a more parsimonious set of parameters, univariate data, and a hundred repetitions, we assessed the resampling risk and speed. Real data was used as an illustration in which speed and resampling risk were also evaluated.

### Synthetic data: Phase I

The dataset consisted of *N* = 20 synthetic images of size 12 × 12 × 12 voxels, containing random variables following either a Gaussian distribution (with zero mean and unit variance) or a Weibull distribution (with scale parameter 1 and shape parameter 1/3, shifted and scaled so as to have expected zero mean and unit variance^3^). The use of these two distributions is to cover a large set of real world problems, with a well-behaved (Gaussian) and a skewed (Weibull) distribution. While the methods are not limited to imaging data, the use of images is helpful for permitting the assessment of the methods using spatial statistics.

To these images, and following the notation from the section Notation and general aspects, simulated effects were added as **M*ψ***, with ***ψ*** = [*ψ*_1_ 0]', *ψ*_1_ being either 0 (no effect) or *t*_cdf_^− 1^(1 − *α*; *N* − rank(**M**))/(**C** ' (**M** ' **M**)^+^** C**)^1/2^, where **C** = [1 0]' is the contrast and *α* = 0.05 is the significance level of the permutation test to be performed at a later stage, thus ensuring a calibrated signal strength sufficient to yield an approximate power of 50% with Gaussian errors, irrespective of the sample size; for the Weibull distribution, the signal was further weakened by a factor 5/8, also ensuring power of approximately 50%. Signal was added to all voxels, thus avoiding the usual problems of signal bleeding, due to smoothing, to areas of otherwise pure noise. The effect was coded in the first regressor only, with the second regressor modelling an intercept. The first regressor was constructed as a set of random values following a Gaussian distribution with zero mean and unit variance. Smoothing was applied with a Gaussian kernel of full width at half maximum (FWHM) of 4 voxels in all three directions, implemented as multiplication in the frequency domain, without zero padding, such that positive dependencies among voxels was introduced as desired, and without producing edge artefacts.

Tests were performed using just one such simulated image (univariate) or three (multivariate data). For the latter, both CMV and NPC test statistics were considered, using Wilks' *λ*, and Pillai's trace for CMV, and the combining functions of Tippett and Fisher for NPC (Winkler et al., 2016). These cover the most common cases. For all these statistics, permutations (for exchangeable errors, EE), sign flippings (for independent and symmetric errors, ISE), and permutations with sign flippings (EE and ISE) were performed. To assess how the parameters needed for each acceleration could impact results, these were varied:

- Few permutations: *J* = {40, 60, 100, 200, 300, 500, 1000, 2000, 5000}, where *J* is the number of permutations.
- Negative binomial: *n* = {2, 5, 10, 15, 20, 50, 100} and *J* = {50000}, where *n* is the number of exceedances before interrupting the process.
- Tail approximation: *J* = {40, 60, 100, 200, 300, 500, 1000, 2000, 5000}, using *p* = 0.10 as the threshold below which the *p*-values are refined, and including or not the first permutation test statistic, *T*_1_^⁎^ ≡ *T* in the initial null distribution to which tail the GPD is fit.
- No permutation: No parameters to be varied for this method.
- Gamma approximation: *J* = {40, 60, 100, 200, 300, 500, 1000, 2000, 5000}, and including or not the first permutation test statistic in the initial null distribution, to which the gamma is fit.
- Low rank matrix completion: *v* = {42, 105, 210, 864} and *J* = {210, 300, 500, 1000, 2000, 5000, 50000}, where *v* is the number of voxels randomly selected to infer the values of all others. The value *v* = 210 corresponds to *v*_0_ = *N*(*N* + 1)/2. We expected that *v* equal to or larger than this critical value would allow perfect reconstruction of the test statistic, but wanted to assess whether smaller values (one half or one fifth of this value) would still be acceptable as approximations; the *v* = 864 corresponds to oversampling. For the univariate case only, a further run using *J* = 50000 and the exact same permutations as the reference set was used to verify their equality.

The 81 possible configurations above generated 709 sets of results considering the univariate, the two CMV, and the two NPC, and the univariate non-spatial statistics (uncorrected and FWER-corrected), TFCE (uncorrected and FWER-corrected) and cluster extent and mass (corrected). Further, the 12 combinations of signal, noise and shuffling strategy required a total of 8508 scenarios to be considered. Each of the six acceleration methods were compared to a reference set produced with *J* = 50000 permutations, which were assessed using PP and QQ plots, constructed in logarithmic scale [henceforth log(PP) and log(QQ)] so as to emphasise the smaller, more interesting *p*-values, and Bland–Altman plots (Bland and Altman, 1986), all with 95% confidence intervals estimated from an approximation to the binomial distribution using the Wilson method (Wilson, 1927). Error rates and power were computed using respectively the simulations without and with signal.

### Synthetic data: Phase II

In addition, for the univariate, Gaussian errors, with and without signal, and exchangeable errors (permutations only), 100 realisations were performed using all the various methods and respective parameters, except low rank matrix completion (Phase I demonstrated it produces identical results as using ordinary permutations; see the Results section). This allowed empirical standard deviations, as opposed to estimated confidence intervals, to be computed and included in the log(PP) and Bland–Altman plots. Histograms of *p*-values, with the variability on the heights of the bars, could also be computed. Estimates of error rates, power, and resampling risk were obtained, as well as elapsed times. These simulations also allowed log(QQ) plots for the extremum statistic, based on the 100 repetitions, as opposed to plots for the corrected FWER *p*-values as in Phase I.

### Real data

We conducted a re-analysis of the data of the voxel-based morphometry (VBM) study by Douaud et al. (2007). In brief, *T*_1_-weighted magnetic resonance images of 25 subjects diagnosed with schizophrenia and 25 controls matched for sex and age were obtained. These images were analysed with FSL-VBM^4^ (Douaud et al., 2007), an optimised VBM protocol (Good et al., 2001) carried out with the FMRIB Software Library (FSL; Smith et al., 2004). In short, the grey matter was segmented from the *T*_1_-weighted image, non-linearly registered to a common space, modulated and smoothed, and the two groups of subjects compared using a design corresponding to a two-sample *t*-test. This is the same dataset used in the original evaluation of TFCE (Smith and Nichols, 2009) and for the present re-analysis, we considered the same two levels of smoothing, i.e., with *σ* = 3, that correspond to FWHM of approximately 7 mm. The overall number of voxels included in this analysis was *V* = 231,259.

The parameters used for the acceleration strategies are the same used for Phase I of the simulations, except that for low rank matrix completion, and considering the *N* = 50, the parameters were held fixed at *v*_0_ = *N*(*N* + 1)/2 = 1275 and *J* = 5000. The reason is that using a smaller *v* would cause the method to fail to recover the non-sampled statistics, even approximately, as the simulations in Phases I and II demonstrated (see the Results section), and varying *J*, once *v* has been fixed, is equivalent to the few permutations method.

## Results

Phase I allowed a comparison between *p*-values obtained from the reference set with those obtained by the various acceleration methods and uncorrected error rates, whereas Phase II allowed an estimation of the familywise error rate after multiple repetitions. The VBM example permitted inspection of the results of a practical example of an imaging modality that offers various statistical challenges, particularly with respect to non-stationarity (Hayasaka et al., 2004, Salimi-Khorshidi et al., 2011) and skewness (Salmond et al., 2002, Viviani et al., 2007). The multiplicity of scenarios resulted in the construction of more than 25 thousand plots and maps, which do not fit the journal format; a selection of a few results would unduly overemphasise certain aspects at the expense of others. Instead, we organised these plots in a browsable set of pages, and packaged them into a single, 1.9 GB file that can be downloaded and browsed locally. This file is deposited for long term preservation and public access at the Research Archive of the Bodleian Libraries (ORA-Data), and it constitutes the Supplementary Material that accompanies this paper, accessible under the Digital Object Identifier (DOI): http://dx.doi.org/10.5287/bodleian:v0wY6e6Y0. The results below make ample reference to this material, and its inspection is encouraged.^5^

Despite the large and multidimensional nature of the simulations and analysis of the real data, both of which considered many possible parameters, and the fact that each method may have strengths under different evaluation metrics, the overall results are generally simple to describe, and are summarised below.

### Error rate

Nearly all methods, when used according to their respective theory, yielded, on average, exact error rates. Evidence for this assertion comes from the log(QQ) plots produced in Phase I, that show *p*-values running along the identity line, or not deviating more than by their respective 95% confidence interval, and the log(PP) and histograms produced from the hundred repetitions performed in Phase II, as shown in the Supplementary Material. A notable exception occurred, for the uncorrected case, if the unpermuted statistic *T*_1_^⁎^ was not included in the null distribution for the gamma and tail approximations, and if less than 500 or 1000 permutations respectively were performed, in which case the error rate was on average above the nominal level. For the corrected, error rates were controlled regardless, and the difference between inclusion or not was negligible. Another exception was, for low rank matrix completion, the use of fewer than the prescribed *v*_0_ tests, which led to error rates being not well controlled; using at least this quantity not only allowed the method to remain exact, but produced results in complete agreement (that is, perfectly identical) to using the same number of permutations and full sampling (that is, without completion).

### Power

Conditional on the error rate being controlled, all methods yielded generally similar power, as evidenced by the histograms in produced in Phase II, shown in the Supplementary Material. It should be noted, however, that although more permutations did not intrinsically increase power, as expected they allowed smaller *p*-values to be found, thus being beneficial for methods that use permutation (few permutations, tail approximation, gamma approximation, and low rank matrix completion) if the significance level were smaller than *α* = 0.05, and certainly for the use of FDR.

### Agreement with the reference set

The smaller *p*-values (e.g., smaller than 0.10), were generally similar across methods, agreeing well with the reference set of results produced with 50000 simple permutations, without considerable variations that would result in entirely different results, both in the presence and absence of signal, although for *p*-values in the middle of the distributions, results often varied widely. In the Supplementary Material, this can observed in the log(PP) and Bland–Altman plots. The two important exceptions were: (I) for low rank matrix completion using fewer tests (voxels) than *v*_0_, that led to widespread disagreement with the reference set and often nonsensical results, and (II) for the no permutation method if the resampling used only sign flipping, or if the errors were skewed. Moreover, for *p*-values away from the tail, the disagreement of the no permutation method with the reference set was substantial, even with symmetric errors and permutations only.

### Resampling risk

**Fig. 2:**
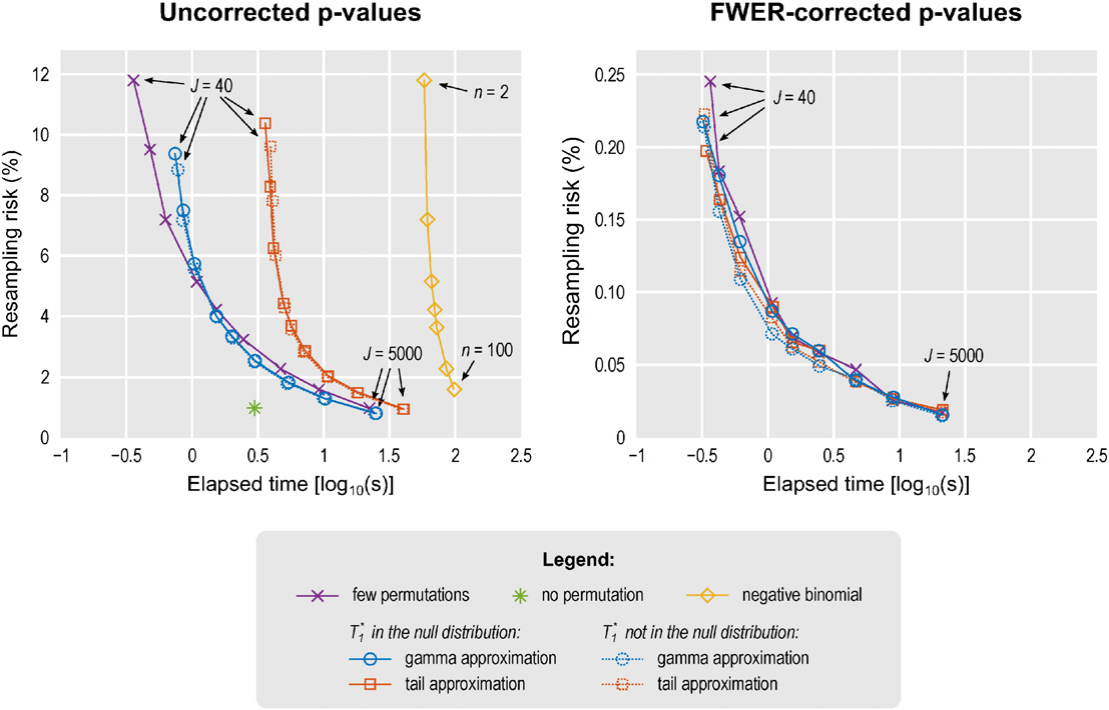
Balance between resampling risk when compared to a reference set of *J* = 50000 permutations and the respective running time, with the data simulated for Phase II (hence, 100 repetitions, Gaussian noise). Some methods have parameters that could be varied: few permutations, tail approximation and gamma approximation use a certain number of permutations that varied in the simulations as *J* = {40,60,100,200,300,500,1000,2000,5000}. The negative binomial distribution uses a fixed upper limit on the number of permutations (set as *J* = 50000) and a number of exceedances that varied as *n* = {2, 5, 10,15,20,50,100}. The no permutation method has no parameter to be varied. The low rank matrix completion has the same resampling risk as the few permutations, but the running time is too dependent on the size of the data, hence is not shown. More permutations reduce the resampling risk, but take longer to run.

The risk of altering decisions about the rejection of null hypotheses was higher when fewer rearrangements were used for methods where *J* was varied. This could be observed in both uncorrected and corrected *p*-values. Removal of *T*_1_^⁎^ in the methods that fit a distribution reduced marginally the resampling risk compared with keeping the unpermuted statistic in the distribution, although making the test invalid; in either case, the resampling risk was always smaller than for using only few permutations, with either uncorrected or FWER-corrected *p*-values. For the negative binomial, resampling risk was higher with fewer exceedances. The method with no permutations yielded the lowest resampling risk overall for the settings assessed. In any case, the resampling risk can be said to have been generally small, and well below 1% for corrected *p*-values in the simulations. Fig. 2 shows the trade-off between speed and resampling risk for the more conservative case in which *T*_1_^⁎^ is included in the permutation distribution.

### Speed

For comparable resampling risks, the method in which no permutations are performed was the absolute fastest. Few permutations, gamma and tail approximations were generally quick, with tail being slower than gamma for the same number of permutations, and gamma slightly slower than few permutations. This considers a voxelwise fit, for uncorrected *p*-values; if only corrected *p*-values are needed, the time needed for the single fit of the GPD or gamma for the distribution of extremum statistic is negligible. The negative binomial and, specially, low rank matrix completion were the slowest. Low rank, however, is expected to perform better in settings where there are more tests to be performed (more voxels) than those used in the simulations and real data, and with a relatively smaller sample size (Table 4).

### Noise distribution and shuffling strategy

The performance of the various methods was similar in terms of error rates, power, resampling risk, and speed, regardless of the errors being Gaussian or Weibull (skewed). However, as expected given its assumptions, the method in which no permutations are used did not produce correct results that could be compared with the reference set if the reference set used sign-flippings (for either error distribution), or if the errors were skewed (regardless of the shuffling strategy, i.e., permutations, sign-flippings, or permutations with sign-flippings).

### Spatial statistics

The behaviour for spatial statistics followed the same trends as for the voxelwise, non-spatial statistics, in terms of error rates, power, agreement with the reference set, and resampling risk.

### Multivariate statistics and non-parametric combination

Likewise, the results for CMV and for NPC followed similar trends as above, with error rates controlled exactly, and yielding similar power as the reference set, as evidenced by the results of Phase I shown in the Supplementary Material.

### Real data

**Fig. 3:**
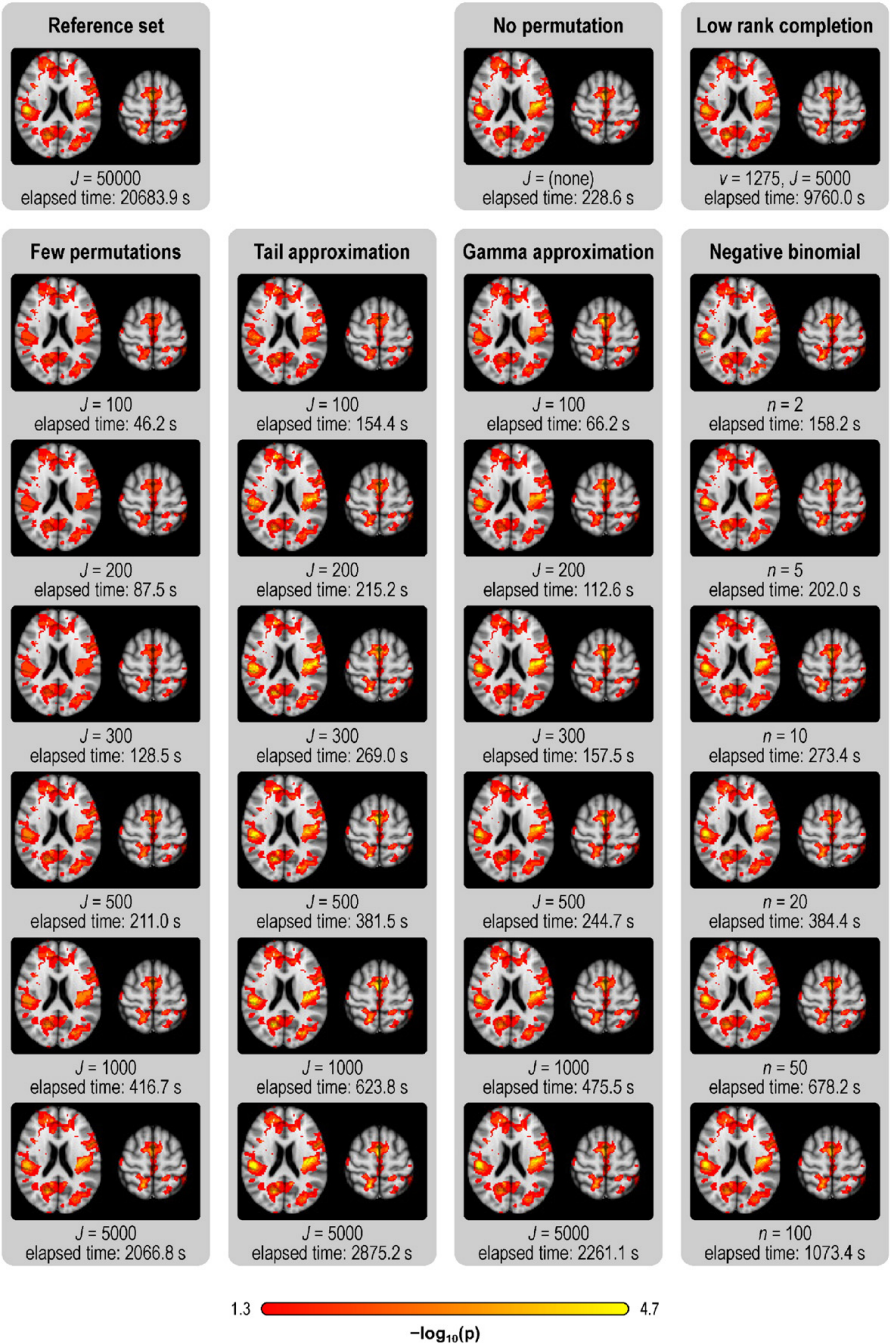
VBM results, showing **uncorrected***p*-value maps (axial slices *z* = 10 and *z* = 48 mm, MNI space), and the overall amount of time taken by each method. The tail and gamma methods generally have higher power compared to few permutations with the same *J*, even with these not including the unpermuted statistic in the null distribution; see the Supplementary Material for other maps.

**Fig. 4:**
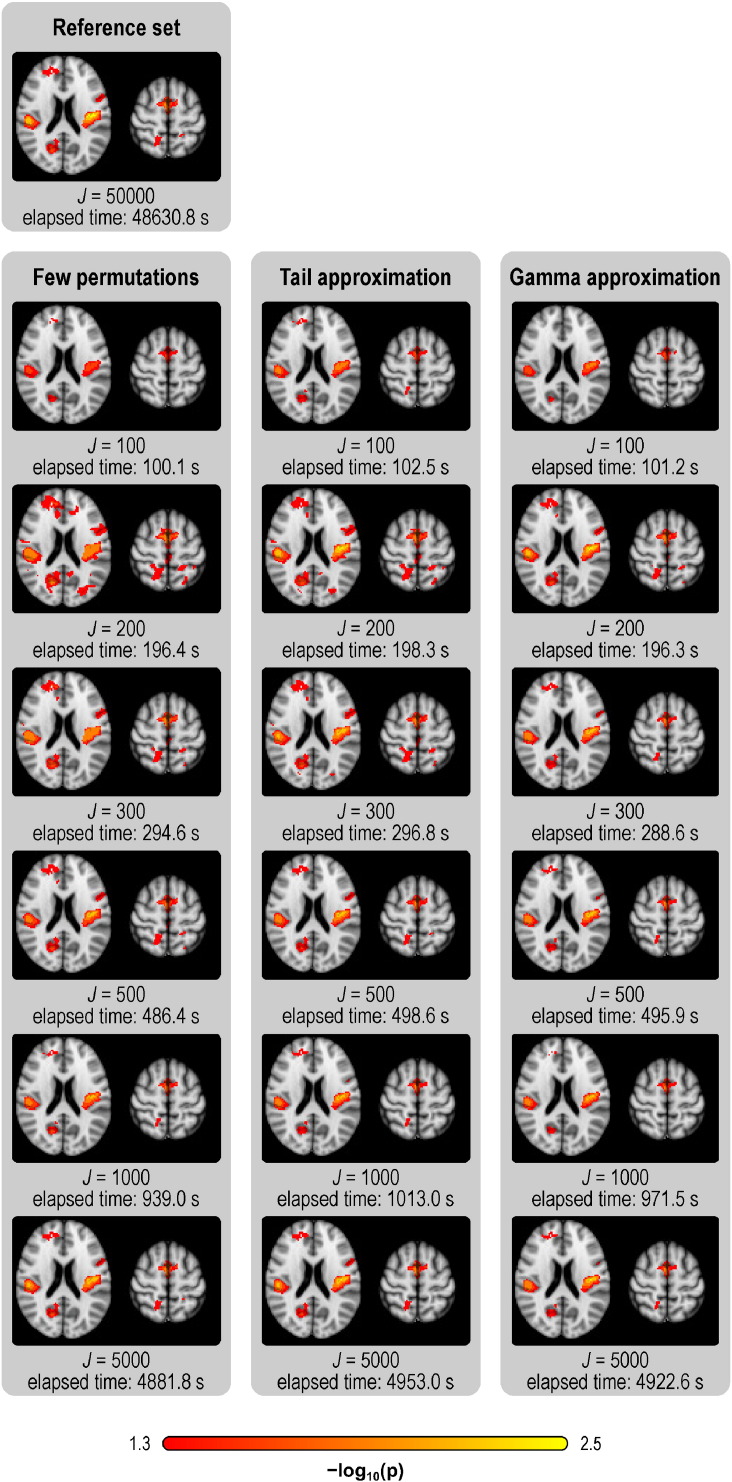
VBM results, showing **corrected** (FWER) *p*-value TFCE maps (axial slices *z* = 10 and *z* = 48 mm, MNI space), and the overall amount of time taken by each method. As with the uncorrected, the methods generally have higher power compared to few permutations with the same *J*, and approximate better the reference set.

All methods yielded visually similar maps for the real data, with smaller *p*-values observable with more permutations for the methods that use permutations, or more exceedances for the negative binomial. In the TFCE, FWER-corrected maps, stronger effects of interest could be revealed by tail and gamma methods for equivalent *J* of few permutations. These results are remarkably similar to the results seen in the reference set, even using about a hundred times fewer permutations, with proportional increases in speed, as summarised in Fig. 3, Fig. 4, and shown in greater detail in the Supplementary Material. The timings refer to the implementation available in PALM, as described at the end of the paper. The acceleration methods worked similarly, and yielded similar increases in speed, for the two levels of smoothing considered.

## Discussion

### Assumptions

All six methods presented are non-parametric in the sense that they do not depend on the distribution of the test statistic. Some of the methods can still be said to be parametric in that certain parameters need to be estimated, such as for the gamma or for the generalised Pareto distribution, although they remain non-parametric in that the distribution from which these parameters are estimated is based on permutations (or at least conceptually, as in the case of the no permutation method). Some methods nevertheless require certain assumptions: for the gamma approximation, a fit can only be adequate if the distribution of the test statistic is unimodal; for the method in which no permutations are performed, the results are an approximation only to permutations proper, not to sign-flippings, and only if the distribution of the errors is symmetric.

### Resampling risk and number of permutations

Although the *p*-values can vary considerably between the methods, as evidenced by the Bland–Altman plots, at the tails they are remarkably similar, thus allowing similar inferences to be drawn, and presenting an overall low resampling risk for the corrected maps. This means that for most methods, the overall result upon rejection or not of the null hypothesis is expected to remain broadly the same.

The results permit relaxing the usual common sense that more permutations are better. Although more permutations do reduce the resampling risk, the high computational cost may not bring additional information upon acceptance or rejection of the null hypothesis, even considering the large number of tests usually performed in brain imaging. This is particularly the case for FWER corrected results, for which the resampling risk, even for moderate to small number of permutations, was quite small.

It should be noted that, although more permutations do not intrinsically increase power, they allow smaller *p*-values to be found (Eq. (3)). Even though *p*-values much smaller than needed to reach a decision on the null hypotheses may be not needed, such as for FWER correction, methods that use uncorrected *p*-values as a starting point for further computations, such as for subsequent FDR correction, stand to benefit more from the greater resolution and potentially greater significance of *p*-values derived with a larger number of permutations. This compounds with more accurate fitting of a distribution, such as the GPD (tail) and gamma, enabled by the larger number of points available in the empirical distribution.

### Tail, gamma, and no permutation

For tail and gamma approximations, a small number of permutations is initially performed, from which a low resolution null distribution is built and used for the GPD (tail) or gamma (full distribution) fit. The results show that inclusion or not of the unpermuted test statistic (*T*_1_^⁎^) in this null distribution makes a substantial difference in the uncorrected case if too few permutations are performed, with *p*-values that, at the tail, are either conservative (if included) or invalid (if not included). Thus, if interest lies solely on uncorrected *p*-values, such as in the absence of multiple testing, or for subsequent use of FDR, other acceleration methods that do not suffer from either conservativeness or invalidity at the tails are advisable. For FWER-corrected *p*-values, as the number of tests (voxels) increase, the difference between including or not the unpermuted statistic in the null distribution becomes negligible.

This is not an unexpected finding, particularly for test statistics that happen to be at the tail, such as when there is a true, strong effect of interest: by being at the tail, *T*_1_^⁎^ is among the rarest values found with the permutations, hence a single extra observation of the statistic is considerably influential if too few permutations are done; for test statistics lying towards the mode of the distribution, where most of the other values are located, a single extra observation has little noticeable effect.

These two methods allow *p*-values to extend further into the tail of the null distribution than otherwise is possible when only few permutations are used, and are particularly useful for the FWER case, offering a complement for the no permutation method that is available to produce uncorrected *p*-values. The latter, however, requires both symmetric error terms and that the intercept is entirely contained in **Z**. Tail and gamma approximation can also be used even if the number of permutations is reasonably large (such as 5000), yielding corrected results that are remarkably similar to what would be obtained with far more shufflings.

### Low rank matrix completion

Various methods can be considered that could make use of low rank matrix completion. The method proposed here performs completion of two matrices, using the data from potentially far fewer tests (voxels) than those present in an image. While completing two matrices, instead of only one, may seem an undesirable computational cost, by restricting the completion to only matrices that can be constructed through linear operations on the data and model, exact recovery is possible. Therefore, problems with unrecoverable residuals due to imperfect reconstruction of the matrix that stores the statistic itself are eschewed, and no assumptions need to be introduced, such as for ad hoc attempts for the recovery of the residuals themselves, or for the characterisation of its parameters. The conditions for completion are easily attainable in brain imaging, and the method produces identical results to those obtained with the conventional permutation test.

The method is expected to perform faster with large images and with small samples, although performance gains also need a fast implementation. The simulations were too expensive to use a sufficiently large image, hence potential advantages of low rank completion could not be illustrated. Yet, the method remains an option as a potential replacement for simple permutations, and as the initial step for tail and gamma approximations. It has also the benefit that, from the recovered statistics, spatial statistics can further be calculated, although direct recovery of such spatial statistics, that are not linear functions of the data, would lead to approximate results.

### Applicability

Most of the assessed methods are generic and can accommodate many cases of potential interest. In particular, the tail and gamma approximations, as well as few permutations, can be applied in a variety of situations that include univariate and multivariate tests (both CMV and NPC), spatial statistics, and for the correction using the distribution of the extremum statistic (minimum or maximum). The low rank matrix completion, by producing identical result to few permutations, can likewise be considered a generic solution, although its computational benefits only arise for large images and with relatively smaller sample sizes, and even so, only for univariate statistics.

Except for the method in which no permutations are performed, all others can be considered for experiments that use non-independent data, as long as dependencies between observations have been taken into account by means of exchangeability blocks, including multiple levels of exchangeability (Winkler et al., 2015), with the consequence that these acceleration methods can be used for experiments that used repeated measurements, heterogeneous variances, or other types of structured dependencies.

### Real data

Using a VBM dataset was especially useful as this imaging method is known to suffer from non-normality, particularly skewness, and spatial non-stationarity, which could pose difficulties. Yet, the acceleration methods performed generally well, and the results of the reanalysis are in line with those of the original study (Douaud et al., 2007). Of note, at *J* = 500, the tail approximation seemed to produce spatial results closer to the reference set than the gamma approximation, with fewer false positives and, importantly, fewer false negatives in relation to that set, especially in the left Broca's area and the inferior temporal gyri. Using of any of the acceleration methods that can produce FWER-corrected *p*-values resulted in the same conclusions about rejection of the null, only with considerable increases in speed. Even though the method in which no permutations are done worked reasonably well with the real and presumably skewed VBM data, it should be noted that assumptions were violated, and this method should not in general be recommended in the presence of skewness.

### Recommendations

As a general rule, given its generalisability, its lack of dependence on symmetry or on unimodality of the permutation distribution, the need to consider the multiplicity of tests in brain imaging, its availability not only for univariate tests, but also CMV and NPC, as well as spatial statistics, and in the absence of any reasonable information about the data, the tail approximation can be recommended. The gamma approximation can be recommended for the same circumstances, and it tends to be slightly faster than the tail approximation, although it requires that the whole permutation distribution is well behaved, and the assumption that its entirety can be approximated by a gamma distribution.

For uncorrected *p*-values, and without spatial statistics, if symmetry of the error terms can be assumed, the method in which no permutations are performed can be recommended, given its speed. If symmetry cannot be assumed, negative binomial distribution and tail approximation can be used; for the latter, the unpermuted statistic may be excluded from the null distribution if the number of permutations is large given the significance level (such as about a thousand for an *α* = 0.05, as considered in the Evaluation), or if the approximation is used for FWER corrected *p*-values. The low rank matrix completion can be considered when the number of tests (voxels) is much larger than the number of subjects, as a replacement to the few permutations, or to build the initial null distribution before tail or gamma approximations.

**Fig. 5:**
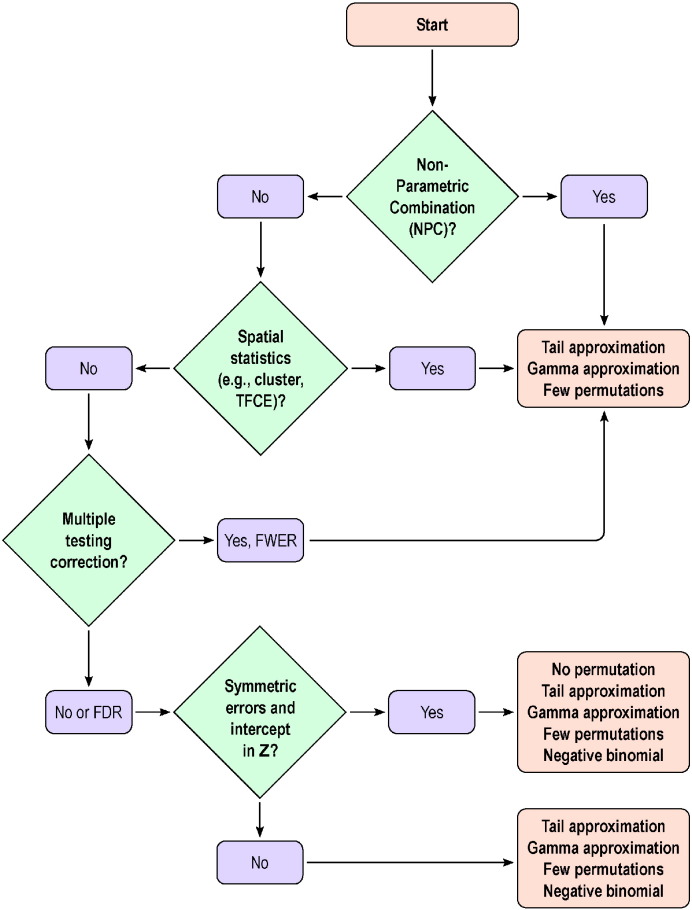
Decision tree regarding the various acceleration methods. Each of the terminal boxes show, in order, the preferred methods. For NPC, spatial statistics, or for FWER-corrected *p*-values, tail and gamma approximations, and few permutations are in general recommendead; gamma is faster than tail fitting, but the latter is more generic. For uncorrected *p*-values, without spatial statistics, and if the errors can be assumed symmetric, the no permutation method is preferred; if symmetry cannot be assumed, the negative binomial is favoured. The low rank matrix completion (not shown) can be used if *N* ≪ *V*, as a replacement to the few permutations or to build the initial null distribution before tail or gamma approximations.

As for the number of shufflings to be used, the choice depends on how small the *p*-value needs to be for a given significance level while maintaining a reasonably small resampling risk. The results seem to indicate that, even without tail or gamma approximations, using about 500 permutations can give stable results for FWER corrected inference, although whenever computational resources are available, more should be considered. The fitting of a GPD or gamma distributions can help with the discreteness that can render FDR conservative. A flow chart summarising these recommendations is shown in Fig. 5.

## Conclusions

A number of statistical devices can be considered to accelerate permutation tests in addition to, or irrespective of, generic improvements to accelerations that depend on software implementation or on hardware. The methods considered yielded generally similar results, and as the different scenarios of error terms and shuffling strategy varied, the methods performed marginally better or worse than each other as assessed in terms of conservativeness, agreement with the reference set, and resampling risk. The methods were in general considerably faster than the common alternative of running a large number of permutations.

Implementation of all the acceleration methods described, licensed under the General Public Licence (GPL), and that can be executed in MATLAB (The MathWorks Inc., 2015) or Octave (Eaton et al., 2015), is available in the tool *Permutation Analysis of Linear Models* (PALM), available for download at www.fmrib.ox.ac.uk/fsl.

## Figures and Tables

**Table 1:** Overview of various strategies that can be considered to accelerate permutation tests.

Method	Brief description	Univariate				CMV				NPC			
		Pointwise		Spatial		Pointwise		Spatial		Pointwise		Spatial	
		unc.	corr.	unc.	corr.	unc.	corr.	unc.	corr.	unc.	corr.	unc.	corr.
Few permutations	Compute the *p*-values using just a few permutations, e.g., less than a thousand.												
Negative binomial	Run for each voxel as many permutations as needed until a predefined number of exceedances is found. Then divide this number of by the number of permutations.												
Tail approximation	Run a small number of permutations and, for the *p*-values below a certain threshold (e.g., 0.10), fit a generalised Pareto distribution, modelling the tail of the permutation distribution.												
No permutation	For statistics that can be written as trace(**AW**), where **A** = **XX**^+^, **W** = **UU**', and **USV** ' = svd(**R**_**Z**_**Y**), compute analytically the moments of the permutation distribution, then fit a gamma distribution.												
Gamma approximation	Run a small number of permutations, compute empirically the moments of the permutation distribution, then fit a gamma distribution.												
Low rank matrix completion	Run a certain number of permutations, define orthonormal bases for matrices that are linear functions of the data and from which the statistic can be obtained; continue permuting a random subset of tests, filling the missing ones via projection to these bases.												

Can be used.   Can be used, although there are particularities (see main text).  Cannot be used.

CMV: Classical multivariate test (such as MANCOVA); NPC: Non-Parametric Combination; see Winkler et al. (2016) for details. Although the tail and gamma approximations can be considered for essentially any permutation distribution (the latter particularly for unimodal distributions), the Results showed that the fit performs better for the distribution of the extremum statistic, as used for familywise error rate (FWER) correction. The negative binomial can be used for NPC, although unlikely with any acceleration benefit. For low rank matrix completion, many algorithmic variants can be considered, and the complexity needed for CMV and NPC may offset speed benefits; for this method, spatial statistics can be computed from the completed non-spatial (pointwise) statistics, although a direct computation, in a similar way as for the pointwise, would require a different algorithm with results that would likely not be exact. See main text for details on this and on all other methods.

**Table 2:** Confidence intervals (95%), computed using the Wilson method, for a *p*-value *P* = 0.05 as a function of the number of permutations (*J*). More permutations narrow the confidence interval.

Number of permutations	Confidence interval
40	0.0138–0.1650
60	0.0171–0.1370
100	0.0215–0.1118
200	0.0274–0.0896
300	0.0305–0.0808
500	0.0341–0.0728
1000	0.0381–0.0653
2000	0.0413–0.0604
5000	0.0443–0.0564
10,000	0.0459–0.0544
50,000	0.0481–0.0519

**Table 3:** A number of methods are available to obtain parameter estimates and construct the permutation distribution in the presence of nuisance variables. Comparative details and references for each of these approaches are in Winkler et al. (2014, Table ~ 2); see also Anderson and Legendre (1999), Anderson and Robinson (2001). For the method of low rank matrix completion, **B** can be written as a product $\tilde{X}\tilde{Y}$, where $\tilde{X}$ is a *J* × *N* matrix that contains the pseudo-inverse of the model on each row, and $\tilde{Y}$ is an *N* × *V* matrix that contains the data. The *j*-th row of $\tilde{X}$ is shown as ${\tilde{x}}_{j}$, whereas the *v*-th column of $\tilde{Y}$ is shown as ${\tilde{y}}_{v}$. The rank(**B**) is at most *N*, and can be smaller for most methods, even when *V* > *N* and *J* > *N*, given the projection to subspaces due to **R**_**Z**_ and **R**_**M**_. The matrix **Σ** has rows ςj=diagY~'RY~, and its rank is, at most, *N*(*N* + 1)/2. This determines the number *J*_0_ of initial permutations to identify an orthonormal basis, and the number *v*_0_ of tests that need to be done to allow exact recovery. See the text for details.

Method	Model	${\tilde{x}}_{j}$	${\tilde{y}}_{v}$	R
Draper–Stoneman	**Y** = **PX*β*** + **Z*γ*** + ***ε***	C~'PjXZ+	**Y**	**I** − [**P**_*j*_**X**, **Z**][**P**_*j*_**X**, **Z**]^+^
Still–White	**PR**_**Z**_**Y** = **X*β*** + ***ε***	**X**^+^** P**_*j*_	**R**_**Z**_**Y**	**I** − **P**'_*j*_**XX**^+^** P**_*j*_
Freedman–Lane	(**PR**_**Z**_ + **H**_**Z**_)**Y** = **X*β*** + **Z*γ*** + ***ε***	C~'XZ+Pj	**R**_**Z**_**Y**	**I** − **P**′_*j*_[**X**, **Z**][**X**, **Z**]^+^** P**_*j*_
Manly	**PY** = **X*β*** + **Z*γ*** + ***ε***	C~'XZ+Pj	**Y**	**I** − **P**′_*j*_[**X**, **Z**][**X**, **Z**]^+^** P**_*j*_
ter Braak	(**PR**_**M**_ + **H**_**M**_)**Y** = **X*β*** + **Z*γ*** + ***ε***	C~'XZ+Pj	**R**_**M**_**Y**	**I** − **P**′_*j*_[**X**, **Z**][**X**, **Z**]^+^** P**_*j*_
Kennedy	**PR**_**Z**_**Y** = **R**_**Z**_**X*β*** + ***ε***	**X**^+^** R**_**Z**_**P**_*j*_	**R**_**Z**_**Y**	**I** − **P**′_*j*_**R**_**Z**_**XX**^+^** R**_**Z**_**P**_*j*_
Huh–Jhun	**PQ** ′ **R**_**Z**_**Y** = **Q** ′ **R**_**Z**_**X*β*** + ***ε***	**X**^+^** R**_**Z**_**Q**′^+^** P**_*j*_	**Q** ′ **R**_**Z**_**Y**	**I** − **P**′_*j*_**Q** ′ **R**_**Z**_**XX**^+^** R**_**Z**_**Q**′^+^** P**_*j*_
Dekker	**Y** = **PR**_**Z**_**X*β*** + **Z*γ*** + ***ε***	C~'PjRZX'Z+	**Y**	**I** − [**P**_*j*_**R**_**Z**_**X**′, **Z**][**P**_*j*_**R**_**Z**_**X**′, **Z**]^+^

While the models as shown can be used for any general linear model (uni or multivariate), here the focus is on the univariate case (*K* = 1 or *Q* = 1) and in which rank(**C**) = 1, such that **Y** and **X** are *N* × 1 matrices (column vectors). After the partitioning, the effective contrast, $\tilde{C}$, is a column vector of length *R*, full of zeroes except for the first element, that is equal to one. **Q** is an *N* × *N*' matrix, where *N*' is the rank of **R**_**Z**_. **Q** is computed through Schur decomposition of **R**_**Z**_, such that **R**_**Z**_ = **QQ**' and **I**_*N* ' × *N*'_ = **Q** ' **Q** (for this method only, **P** is *N* ' × *N*'; otherwise it is *N* × *N*). **R**_**M**_ = **I**_*N* × *N*_ − **MM**^+^. All other variables are described in the text. (It has been brought to our attention that the Smith method cited in Winkler et al. (2014) had been proposed previously by Dekker et al. (2007), hence it is here renamed.)

**Table 4:** Computational complexity and memory requirements for the different methods.

Method	Computational complexity	Specific storage
Few permutations	Θ(*NVJ*)	2*V*
Negative binomial	Θ(*nN* log (*V*))	2*V*
Tail approximation	Θ(*V*(*NJ* + 1))	*V*(*J* + 1)
No permutation	Θ(*NV*)	V
Gamma approximation	Θ(*V*(*NJ* + 1))	*V*(*J* + 1)
Low rank matrix completion	Θ(*N*^3^(*V* + *J*))	2*V*(2*J*_0_ + 1)

*N* is the sample size, *V* the number of tests in an image (such as voxels or vertices), *n* the number of exceedances, and *J* the number of permutations, and *J*_0_ the number of fully sample permutations in the low rank matrix completion method. The computational complexity refers to the acceleration, and does not include steps that are common to all methods, such as the model partitioning, computation of the test statistic and other procedures. Likewise, the specific storage refers to the amount of memory needed to store the bulk of the intermediate data that are particular for each method, and ignores storage needs that are common to all methods, such as for the data itself, the design matrix, the set of permutations, etc.; it also ignores small transitory variables that occupy insignificant amounts of memory. Tail and gamma as indicated consider the fitting for uncorrected *p*-values, that need one fit per test (voxel); if only FWER is required, the cost of a single fit is negligible, and these can be considered Θ(*NVJ*).

## Footnotes

1 The shape parameter *ξ* of the GPD corresponds to the shape parameter of the generalised extreme value distribution, whereas the scale parameter *σ* relates to the GEV scale *s* as *σ* = *s* − *ξ*(*u* − *μ*), where *μ* is the GEV location parameter.

2 To see this, let $H=\left(C'\hat{\psi }D\right)'{\left(C'{\left(M'M\right)}^{-1}C\right)}^{-1}\left(C'\hat{\psi }D\right)$ and $E=\left(\hat{\varepsilon }D\right)'\left(\hat{\varepsilon }D\right)$ be, respectively, the sums of products explained by the model (hypothesis) and the sums of the products of the residuals, i.e., that remain unexplained. Pillai's statistic is *T* = trace(**H**(**H** + **E**)^− 1^). With the model simplification and partitioning, H=Xβ^'Xβ^=HXY~'HXY~=Y~'HXY~ and E=RXY~'RXY~=Y~'RXY~, where **R**_**X**_ = **I** − **H**_**X**_. Thus, H+E=Y~'HX+RXY~=Y~'Y~. The trace of a product is invariant to a circular permutation of the factors, such that T=traceY~'HXY~Y~'Y~−1=traceHXY~Y~'Y~−1Y~'=traceHXY~Y~+. Using the factors of a singular value decomposition, Y~=USV' and ${\tilde{Y}}^{+}=VS^{+}U'$, where **U** contains only the K columns that correspond to non-zero singular values, the statistic becomes *T* = trace(**H**_**X**_**USV** ' **VS**^+^** U**') = trace(**H**_**X**_**UU**'). The matrices **H**_**X**_, **UU**', and $\tilde{Y}{\tilde{Y}}^{+}$ are *N* × *N*.

3 Thus with actual skewness (Γ(1 + 3/*k*)*λ*^3^ − 3*μσ*^2^ − *μ*^3^)/*σ*^3^ ≈ 19.58, where here *μ* and *σ*^2^ represent the mean and variance of this distribution, and *k* and *λ* the shape and scale parameters.

4 Available at http://fsl.fmrib.ox.ac.uk/fsl/fslwiki/fslvbm.

5 A mirrored copy that does not require download, though not guaranteed for permanent preservation, can be found at http://fsl.fmrib.ox.ac.uk/analysis/fastpval
